# Virtual Screening of Phytochemicals by Targeting HR1 Domain of SARS-CoV-2 S Protein: Molecular Docking, Molecular Dynamics Simulations, and DFT Studies

**DOI:** 10.1155/2021/6661191

**Published:** 2021-05-20

**Authors:** Arshia Majeed, Waqar Hussain, Farkhanda Yasmin, Ammara Akhtar, Nouman Rasool

**Affiliations:** ^1^Medicare Health Services, Lahore, Pakistan; ^2^National Center of Artificial Intelligence, Punjab University College of Information Technology, University of the Punjab, Lahore, Pakistan; ^3^Center for Professional & Applied Studies, Lahore, Pakistan; ^4^Department of Biotechnology, Khawaja Fareed University of Science and Technology, Rahim Yar Khan, Pakistan; ^5^Department of Life Sciences, University of Management and Technology, Lahore, Pakistan 54770

## Abstract

The recent COVID-19 pandemic has impacted nearly the whole world due to its high morbidity and mortality rate. Thus, scientists around the globe are working to find potent drugs and designing an effective vaccine against COVID-19. Phytochemicals from medicinal plants are known to have a long history for the treatment of various pathogens and infections; thus, keeping this in mind, this study was performed to explore the potential of different phytochemicals as candidate inhibitors of the HR1 domain in SARS-CoV-2 spike protein by using computer-aided drug discovery methods. Initially, the pharmacological assessment was performed to study the drug-likeness properties of the phytochemicals for their safe human administration. Suitable compounds were subjected to molecular docking to screen strongly binding phytochemicals with HR1 while the stability of ligand binding was analyzed using molecular dynamics simulations. Quantum computation-based density functional theory (DFT) analysis was constituted to analyze the reactivity of these compounds with the receptor. Through analysis, 108 phytochemicals passed the pharmacological assessment and upon docking of these 108 phytochemicals, 36 were screened passing a threshold of -8.5 kcal/mol. After analyzing stability and reactivity, 5 phytochemicals, i.e., SilybinC, Isopomiferin, Lycopene, SilydianinB, and Silydianin are identified as novel and potent candidates for the inhibition of HR1 domain in SARS-CoV-2 spike protein. Based on these results, it is concluded that these compounds can play an important role in the design and development of a drug against COVID-19, after an exhaustive *in vitro* and *in vivo* examination of these compounds, in future.

## 1. Introduction

The COVID-19 pandemic was reported initially in December 2019 in Wuhan, a populous city of China. Till now, it is spreading viciously around the globe affecting the lives of millions of people [1]. Until now, millions of people have been diagnosed with the COVID-19 infection, along with high mortality rate, as it is being updated daily by WHO and other authorities (https://www.worldometers.info/coronavirus/). Although there are many speculations associated with the emergence of the virus, the most authentic source suggests the emergence of the virus from the wild animal (bats, civets, and pangolin) market in Wuhan city. There exist various vaccines till date; however, the effectiveness of these vaccines is still under question, which depicts that the world is in desperate need of anti-COVID-19 drugs and proper remedies [2]. For treatment, scientists are experimenting with a combination of different antiviral drugs. Along with these drugs, doctors are also utilizing the neutralizing antibodies associated with the SARS and MERS-related coronavirus mainly targeting the spike protein of SARS-CoV-2. Like other coronaviruses, the spike protein of SARS-CoV-2 is also considered a prime target for the designing of effective inhibitory drugs against this coronavirus infection [3].

**Figure 1:**
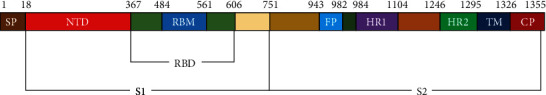
Illustration of the spike protein and its all regions.

SARS-CoV-2 is predominantly a member of the *beta coronaviruses*, which has been previously reported with the name of other flu-like infections (SARS and MERS). These viruses were initially thought to be only infecting wild animals until, in the year 2002, when the SARS outbreak was first reported in the Chinese city, Guangdong [4]. A decade later, the MERS outbreak was reported in the ME (Middle East) countries [5]. Coronaviruses are mainly divided into *α* (alpha consisting of NL63 and Human alphacoronavirus 229E), *β* (beta), gamma (*γ*), and delta (*δ*) coronavirus. The coronaviruses are minuscule viruses, ranging in size from 65 to 125 nm, and are single-stranded RNA viruses. These viruses consist of structural proteins which are spike (S), nucleocapsid (N), matrix protein (N), and envelope (E) as well as a set of nonstructural proteins, for example, nsp5, nsp12, and nsp3. The spike glycoproteins are the type of fusion proteins that play an integral role in viral attachment through the phenomena of recognition and its ultimate entry into the host cells. The spike protein is present on the outer surface of the SARS-CoV-2 in the homotrimeric form and mediates binding with the ACE2 human receptor, thus providing an entry of the virus into the lungs [6, 7]. The attachment is done with the help of the S1 subunit of the spike glycoprotein. Through the binding domain, after binding, the spike protein alters its confirmation for fusion and internalization of the virus in the host cell [8]. The viral fusogenic mechanism of SARS-CoV-2 mainly depends on the interaction of heptad repeat 1 and 2 (HR1 and HR2) regions in the spike protein [9]. Furthermore, Huang et al. [10] reported antibodies based fusion inhibitors of the HR1 domain for the treatment of SARS-CoV-2 and identified EK1, which is a fusion inhibitor and predominantly targets the HR1 domain of SARS-CoV-2. This highlights the importance of the HR1 domain and depicts that more work is needed to be done by targeting the HR1 domain of SARS-CoV-2, to find potent viral inhibitors [11]. Heptad repeat 1 and 2 (HR1 and HR2) regions from spike protein and overall structure of spike protein are shown in Figure 1.

Phytochemicals have been known from ancient time for their immense potential and tremendous properties against several infectious diseases and health-related complications [12]. The phytochemicals constitute magnificent antiviral potentials and can be used in treating viral infections, for example, Liu et al. [13] documented the magnificent antiviral activities of silymarin compounds against several viruses including hepatitis and influenza viruses. With time, *in silico* approaches are gaining much attention around the world for their advanced strategies and effective techniques related to the field of medical sciences. Various previously reported studies have been based on computer-aided protocols for drug discovery, which further help in screening identifying compounds, so that those compounds can be further led to in vitro and in vivo assessments [14, 15]. Computer-aided strategies provide an operative platform, where scientists can analyze a wide range of biological phenomena, different pathways, and molecular interactions. These methods are primarily cost-effective and consist of authentic methods which predict the results with the highest accuracy because, when the number of compounds to be analyzed is high, opting for biological validations can be hectic and very expensive [16–18]. They fall in the dry lab category and are widely used before experimenting in the scientific laboratory [19].

In this study, the HR1 domain in SARS-CoV-2 spike protein was targeted using a set of 2750 phytochemicals to explore their inhibitory potential as effective inhibitors for the treatment of novel coronavirus in future. Absorption, distribution, metabolism, excretion, and toxicity (ADMET) of the phytochemicals were analyzed to study their pharmacological properties and pharmacokinetics. The docking methods were employed to ascertain the binding interactions of the compounds with the targeted protein, and binding stability was analyzed using molecular dynamics (MD) simulations. Finally, quantum computation-based density functional theory (DFT) analysis was constituted to analyze the reactivity of these compounds with the target.

## 2. Material and Methods

### 2.1. Collection and Preparation of Ligands and Receptor

A total of 2750 phytochemicals, from 463 plants, were discerned by carrying out detailed searching from different databases and literature sites, based on their medicinal properties. The origin targeted for plants was Pakistan and India, and the list of plants is provided in Table S2. An extensive literature review was performed to initially search out those plants which belonged to the subcontinent and later to search out the phytoconstituents of that specific plant. In this way, a list of plants and their phytochemicals was screened while the 3D structures of ligands were downloaded from PubChem in SDF format and later converted into PDB by using Chimera. For those, whose structure was not available in PubChem, the structures were sketched manually in ACD ChemSketch [20–26]. The set comprised of a variety of phytochemical groups, i.e., 476 Alkaloids, 312 Terpenoids, 110 Aurones, 128 Chalcones, 402 Flavonoids, 215 Lignans, 340 Carboxylic acids, 310 Polyphenols, and 457 others [24]. The protein structure was downloaded from RCSB under the PDB ID: 6VSB, which was the structure of spike glycoprotein of SARS-CoV-2, while for docking, the grid was designed targeting HR1 domain (912-984 residues), only. Dimensions of grid were 32 × 30 × 48 Å^3^ with center at *x* = 208.481, *y* = 235.694, and *z* = 208.301. The binding pocket was analyzed using MetaPocket [27]. The compounds and protein structure were converted into PDBQT format to further analyze and perform docking.

### 2.2. Predicting Pharmacological Properties of Ligand Set

The ADMET analysis of all the compounds was executed by using a web-based server named PreADMET (https://preadmet.bmdrc.kr/) [28]. The drug-likeness properties of all the ligand compounds were analyzed by PreADMET, and ADMET profiling was prepared. All the important parameters associated with the drug-likeness properties of the compounds like Lipinski's rule of five and pharmacokinetics features were computed. The criteria set for screening compounds were as follows: Lipinski′s violations = 0; solubility = high; GI absorption = high or moderate; BBB − permeability = no; toxicity = zero/Nil. Compounds, which violated the screening criteria, were not considered for further analysis. For example, a good drug should be soluble and exhibit no BBB-permeability. The BBB act as a barrier and prevent the entry of any drug into the brain.

### 2.3. Molecular Docking and Binding Analysis

The possible inhibitory effects of these naturally occurring compounds were evaluated by the docking method against the HR1 domain. The AutoDock Tools and AutoDock Vina were utilized for docking protein and ligands [29–31]. Before docking, ligands and protein were prepared by adding polar hydrogens and torsion adjustment specifically for ligands. The estimation of the binding energies was investigated along with compound interaction, and *K*_*i*_ values were also calculated by the equation (1).

(1) $$\begin{array}{c} K_{i}=\frac{∆G}{e^{R\times T}}, \end{array}$$

where Δ*G* is docking energy, *T* stands for temperature that is 298.15 k, and *R* is a gas constant with 1.9872036 kcal/mol of value. *K*_*i*_ is the inhibitory constant which primarily tells about the ability of a compound as a potential inhibitor. To find out the binding affinity of inhibitors with targeted proteins, docking was performed. A grid box was generated with the help of AutoDock Tools, and sizes of *x*, *y*, and *z* were determined. Grid box dimensions for the HR1 domain from 6VSB were 32 × 30 × 48. The binding affinity values of these ligands were determined to evaluate how well they interact with the protein of interest. Docking simulations were carried out with 6 different exhaustiveness heuristics, i.e., *E* = 4, *E* = 8, *E* = 16, *E* = 32, *E* = 64, and *E* = 128. However, no improvement was observed in terms of binding after the exhaustiveness *E* = 8. The output files obtained from docking were used for structural analysis in the Discovery Studio 2.5 [32], where 2D and 3D structural images of binding interaction were generated and visualized.

Constant temperature MD simulations were performed to study the stability in the binding of phytochemicals with target protein using Groningen Machine for Chemical Simulations (GROMACS) v 5.0 [33]. Only those complexes were analyzed, where phytochemicals showed high binding affinity. For all those protein-ligand complexes, the optimized potential for liquid simulation (OPLS-AA) was applied and the system was solvated with spc216 water molecules. This solvated system was neutralized by adding counter ions of Na^+^ and Cl^−^. At the next step, this system was subjected to energy minimization with the steepest descent method, keeping the step limit as 50000. Later on, constant number volume and temperature (NVT) and constant number pressure and temperature (NPT) equilibrations were performed with 1 atm pressure and four different temperatures, i.e., 300 K, 325 K, 340 K, and 350 K. Explicit water molecules were also added in the phosphoserine sites, and for all simulations, standard pH of 7.0 was considered. This set of constraints was selected due to keeping the simulations similar to the human biological system. The duration for both equilibrations was 1 ns whereas the force field used in both equilibrations was Particle Mesh Ewald (PME) with a cubic interpolation implementation [34]. While performing equilibrations, the hydrogen bonds were readjusted with the help of the Linear Constraint Solver (LINCS) technique [35]. The final production MD simulation was performed for 20 ns, keeping the method the same as equilibrations. The analysis of the results was performed using *rms* and *gyrate* utilities of the GROMACS. Based on RMSD, the graph was plotted using Graphing Advanced Computation and Exploration (GRACE) of data [36].

### 2.4. Reactivity Studies through DFT

Density functional theory (DFT) analysis was performed to study the phytochemical reactivity properties and how efficiently they act when used against the HR1 domain. The study was performed by using HOMO (highest occupied molecular orbital) and LUMO (lowest unoccupied molecular orbital) energies. The Δ*E* (band energy gap) calculation was performed using the expression *E*_LUMO_ − *E*_HOMO_. These descriptors are based on quantum mechanics and their computations. The Avogadro was used to prepare the input file which is a chemical analyzer tool, and all the energy calculations were performed using the program named ORCA [37]. For these DFT-based calculations, B3LYP exchange-correlation functional was employed, which is a hybrid exchange-correlation functional. Generally, the hybrid exchange-correlation is a combination of Hartree-Fock exact exchange functional and any other density functional. However, the targeted correlation, i.e., B3LYP is defined as follows:

(2) $$\begin{array}{c} E_{xc}^{\text{B}3\text{LYP}}=E_{x}^{\text{LDA}}+a_{0}\left(E_{\text{x}}^{\text{HF}}-E_{x}^{\text{LDA}}\right)+a_{x}\left(E_{x}^{\text{GGA}}-E_{x}^{\text{LDA}}\right)+E_{c}^{\text{LDA}}+a_{c}\left(E_{c}^{\text{GGA}}-E_{c}^{\text{LDA}}\right), \end{array}$$

where  *a*_0_ = 0.20, *a*_*x*_ = 0.72, and *a*_*c*_ = 0.81. *E*_*x*_^GGA^ is the generalized gradient approximation for the Becke 88 functional while the *E*_*c*_^GGA^ reflects the correlation functional of Lee-Yang-Parr. With this hybrid functional, local density approximation is added in the form of *E*_*c*_^LDA^ [38]. def2-SV (P) basis set was used for the study.

## 3. Results and Discussion

Coronaviruses are zoonotic pathogens and belong to the family Coronaviridae. The current outbreak of coronavirus in the form of SARS-CoV-2, a new strain, is posing an overwhelming impact around the globe. In this hour of need, the discovery of potent drugs and inhibitors against COVID-19 as soon as possible is a formidable challenge for the scientist. Scientist around the world is struggling to find an immediate cure against this infectious disease to lessen the associated disastrous impact globally [39].

### 3.1. ADMET Results

In this study, the potential of phytochemicals was observed against the HR1 domain in SARS-CoV-2 spike protein. A total of 2750 naturally occurring compounds were first inspected for their drug-likeness properties. ADMET analysis is primarily carried out to identify the potentials of these compounds to be used as a human drug in future during clinical experiments [18, 23, 26, 40–44]. Out of 2750 compounds, 1689 were selected based on Lipinski's rule of five. These phytochemicals were further screened based on BBB permeability. The 756 compounds were obtained which showed the characteristics of non-BBB permeability. Other parameters like GI absorption and solubility properties of these phytochemicals were also apprehended. Toxicity and carcinogenic attributes associated with these natural compounds were also enumerated, and in such a way, a total of 108 compounds were obtained after a rigorous ADMET analysis based on their high drug-likeness properties (Table S1). These 108 phytochemicals were docked against the targeted HR1 domain in SARS-CoV-2.

### 3.2. Molecular Docking Screening Results

In computer-aided drug designing approaches, molecular docking plays a fundamental role in selecting the lead compounds. Molecular docking is particularly employed to identify and analyze a different kind of protein-ligand binding interactions. The binding interactions are mainly studied between the drug and the active site of the target protein. The binding energy values provide information about how well and competitively a ligand interacts with the target protein [8]. In this study, the HR1 domain in the SARS-CoV-2 was docked using 108 phytochemicals. All the phytochemicals exhibited good binding properties with the target protein. Out of 108 compounds, 36 were screened after applying the threshold criteria of -8.5 kcal/mol. The compounds which showed binding affinity value greater than or equal to -8.5 kcal/mol were selected as they exhibited promising characteristics for their prospective selection as HR1 domain inhibition candidates. However, the binding affinities of all 108 phytochemicals have been reported in Table S3. The reason for choosing a threshold of -8.5 kcal/mol was that remaining all other compounds showed a significant gap in binding energies, and as after -8.5 kcal/mol, we observed a binding affinity of -7.9 kcal/mol (Table S3). Usually, the threshold is selected based on comparing it with previously reported and biologically validated known inhibitors. But as in our case, no previously reported and biologically validated known inhibitors were present, and a threshold of -8.5 kcal/mol was chosen so that we do not have to further assess all compounds and a reasonable number of compounds can be selected. A similar approach has been opted in various previously reported studies [18, 23, 26, 40, 41, 44].

The phytochemical named SilybinC exhibited the highest binding affinity value of -10.0 kcal/mol (*K*_*i*_ = 0.046 *μ*M) among all the phytochemicals. The compound which exhibited a high binding affinity value after SilybinC was Isopomiferin (-9.9 kcal/mol, *K*_*i*_ = 0.054 *μ*M), which is a derivative of Pomiferin, primarily extracted from *Maclura pomifera.* The compound has been known to exhibit massive antiviral activity [45]. Pomiferin mainly constitutes a strong antioxidant activity, antimicrobial properties, antitumour, and antidiabetic characteristics [46].

Other compounds, which showed strong binding with HR1 domain, mainly included Lycopene = −9.8 kcal/mol, SilydianinB = −9.8 kcal/mol, Silydianin = −9.7 kcal/mol, and Osajin = −9.5 kcal/mol. Lycopene, a tetrapene extracted from *Silybum Marianum*, is a red-coloured compound carotene, mostly found in tomato, and other fruits and vegetables have been known for its immense antioxidant properties [47]. In this study, Lycopene exhibited strong binding interaction with the HR1 domain, showing potential against SARS-CoV-2. Overall, the selected compounds obtained through docking analysis in this study hold an immense potential to act as inhibitors of the HR1 domain in SARS-CoV-2 spike protein. The detailed results are presented in Table 1.

### 3.3. MD Simulation Analysis

At this step, the complexes with screened 36 compounds in the previous phase were subjected to molecular dynamics simulations, whereas these simulations were performed to analyze the stability in complexes and binding of protein and ligands. The radius of gyrations (*R*_*g*_) was plotted to analyze the stability in complexes, while the root means square deviation (RMSD) values were also observed for whole simulations. Here, at this step, the 36 compounds were further screened based on the MD simulation results (RMSD values and *R*_*g*_ fluctuation intervals), and the top five strongly binding phytochemicals were further screened. Average RMSD values are reported in Table 2.

**Figure 2:**
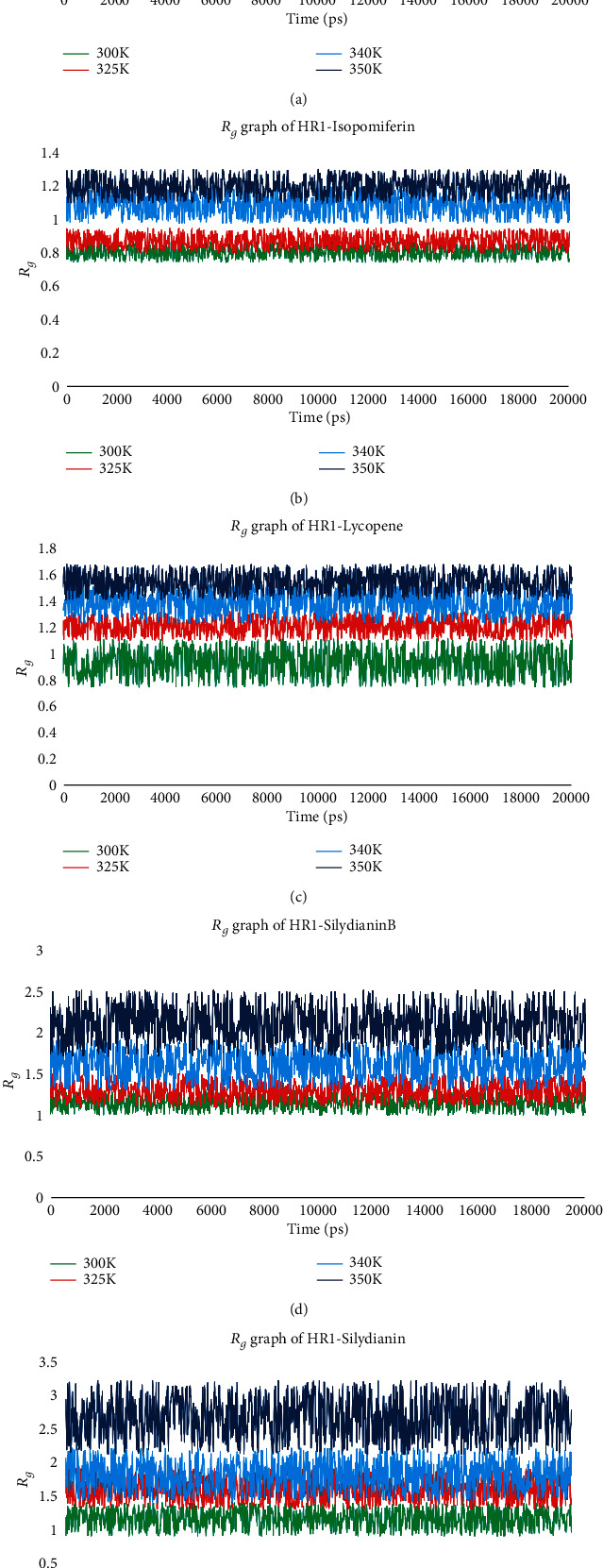
*R*_*g*_ graphs for complexes of strongly binding phytochemicals with HR1 domain. (a) SilybinC, (b) Isopomiferin, (c) Lycopene, (d) SilydianinB, and (e) Silydianin.

According to the results shown in Table 2, the RMSD values were observed to have substantial changes while looking in complexes of HR1 with other phytochemicals; however, within these five complexes, these values were very low, i.e., less than 3 Å [18, 44]. Furthermore, the graphs shown in Figure 2 depicted fewer fluctuations in the radius of gyration. These values depict high stability, compactness, and stable folding of protein tertiary structure, as well as stability in protein-ligand complexes. Values of RMSD increased with the temperature rise, as shown in Table 2, while fluctuations tended to increase in graphs of the radius of gyration. However, the changes were observed in all complexes and not in any specific complex. The increase in temperature caused a decrease in stability and compactness, which leads to such changes. Furthermore, these results are also in accordance with previously reported studies [48, 49].

### 3.4. DFT Results

Density functional theory (DFT) is an advanced *in silico* approach which is progressively gaining popularity in the field of computational-based analysis and works on the principles of quantum mechanics and its descriptors. The study of the electronic properties of the phytochemicals can play a conclusive role in estimating the pharmacological characteristics of these compounds. During analysis, HOMO and LUMO descriptors were computed by utilizing ORCA, whereas the band energy gaps were computed as the difference between *E*_LUMO_ and *E*_HOMO_. The results of DFT-based computations for molecular orbital energy descriptors and band energy gap are shown in Table 3.

The band energy gap values for these phytochemicals ranged from 0.114 kcal/mol to 0.127 kcal/mol, showing narrow energy gaps and proving their high reactivity properties. The result showed that the phytochemicals with a broad range of chemical diversity exhibited good binding interactions with the protein, and the screened phytochemicals exhibited high reactivity. The band energy gaps were computed with the purpose that they are directly associated with the reactivity of compounds, as shown in [50], because the lower band energy gaps depict high reactivity.

**Figure 3:**
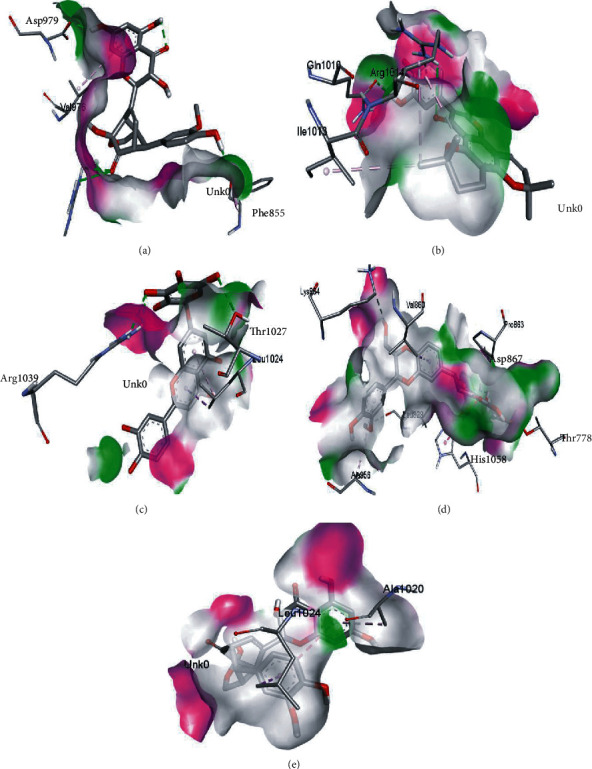
Illustration of the interaction of top five phytochemicals in terms of highest binding affinity, strong binding stability, and reactivity values with HR1 domain. (a) SilybinC, (b) Isopomiferin, (c) Lycopene, (d) SilydianinB, and (e) Silydianin. The purple colour exhibits a hydrogen bond donor, and the green colour shows hydrogen bond acceptor interactions between the HR1 residues and ligand.

The values of *E*_LUMO_ and *E*_HOMO_ were also low, exhibiting the fact that lower band energy gaps result in the high reactivity of the inhibitors for the targeted protein. B3LYP function from DFT was applied to analyze the molecular orbital energies. It is well established in the literature that the lower band energy gap reflects higher reactivity of compounds as the *E*_LUMO_ and *E*_HOMO_ are responsible for the charges transferred in a chemical reaction [51–53]. Both energies can also characterize the electrophilic or nucleophilic nature of a compound. In the present study, the screened phytochemicals were evaluated based upon DFT analysis and the lower band energy gap of molecular orbital energies illustrated the higher reactivity of these phytochemicals, which is in accordance with previously reported studies as well [20–24, 26, 38, 49, 54–56]. The interactions of these top five phytochemicals in the binding pocket of the HR1 domain are shown in Figure 3.

## 4. Conclusion

Due to the recent pandemic of COVID-19 around the globe, caused by SARS-CoV-2, there is a need for potent drugs or vaccines for the treatment of this infection. The use of computational approaches to find novel drug candidates against COVID-19 is an optimal therapeutic approach. In this study, the phytochemicals were tested against the HR1 domain in the spike protein of SARS-CoV-2 to identify novel inhibitors. Based on molecular docking results, it was observed that a total of 36 compounds with the binding affinities crossing the threshold of -8.5 kcal/mol were initially screened while after complete analyses performed in this study, SilybinC, Isopomiferin, Lycopene, SilydianinB, and Silydianin are screened based on the highest binding affinity, strong binding stability, and reactivity scores with the HR1 domain. These compounds exhibited immense potential for their future use as anti-COVID-19 drugs. Hence, these five compounds can be considered as candidate inhibitors for targeted protein and as drugs for human administration, after their thorough *in vitro* and *in vivo* examinations, and clinical trials, in future.

## Figures and Tables

**Table 1:** Binding energies (kcal/mol) and *K*_*i*_ (*μ*M) of compounds with 2-dimensional plots of ligand-protein interaction.

S. No.	Compounds	Binding affinity (kcal/mol)	*K*_*i*_ (*μ*M)	Interaction plots
1.	SilybinC	-10.0	0.046	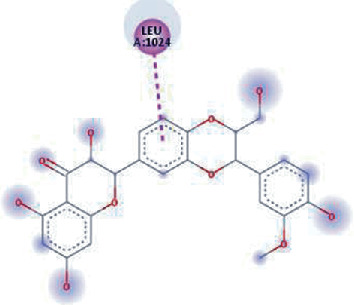
2.	Isopomiferin	-9.9	0.054	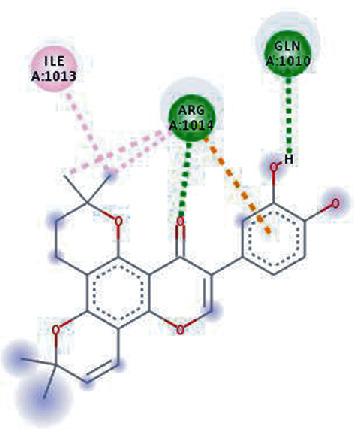
3.	Lycopene	-9.8	0.064	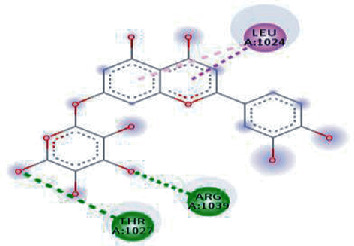
4.	SilydianinB	-9.8	0.064	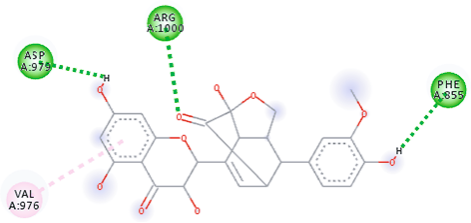
5.	Silydianin	-9.7	0.076	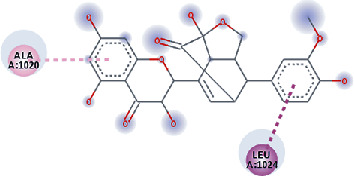
6.	Osajin	-9.5	0.107	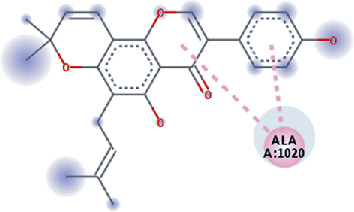
7.	Anthraxin	-9.5	0.107	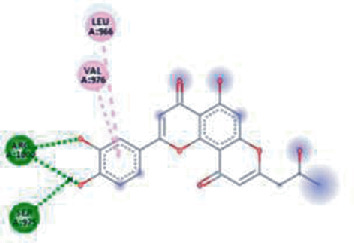
8.	Derrisin	-9.5	0.107	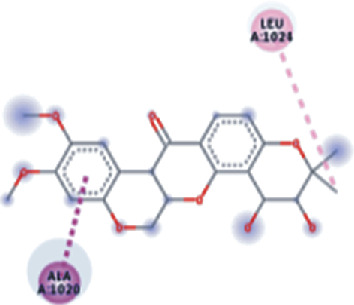
9.	SigmoidinA	-9.4	0.127	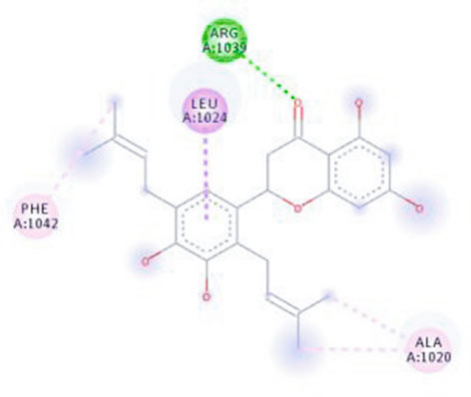
10.	SigmoidinC	-9.4	0.127	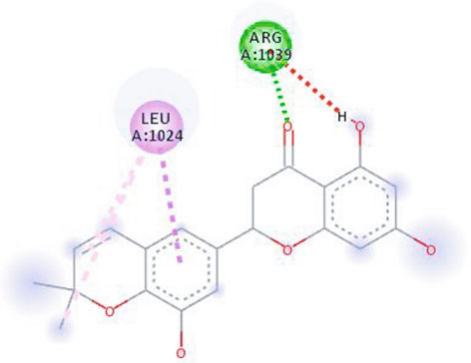
11.	EuchrenoneB	-9.3	0.150	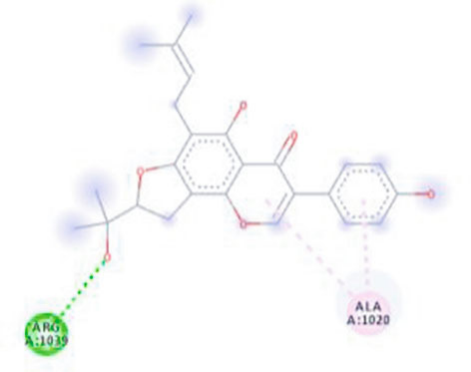
12.	SilybinD	-9.3	0.150	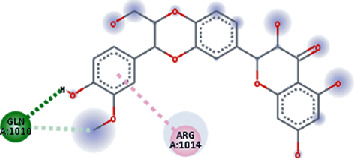
13.	IsosilybinA	-9.2	0.177	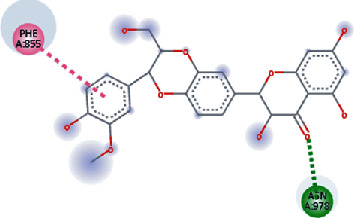
14.	Cannflavin	-9.2	0.177	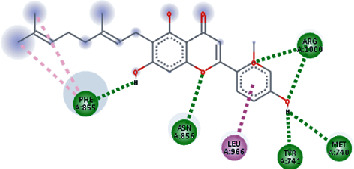
15.	Fumaritine N-oxide	-9.1	0.210	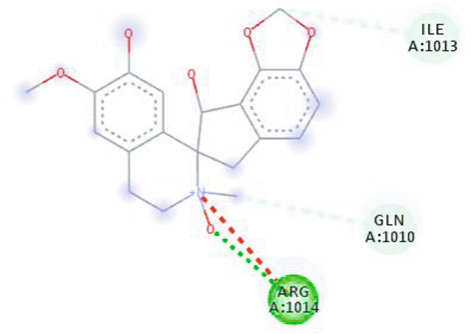
16.	SilybinA	-9.1	0.210	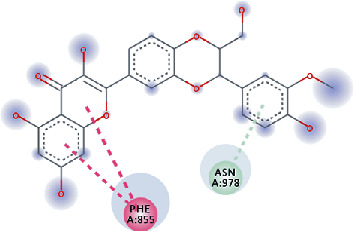
17.	Diprenyleriodictyol	-9.1	0.210	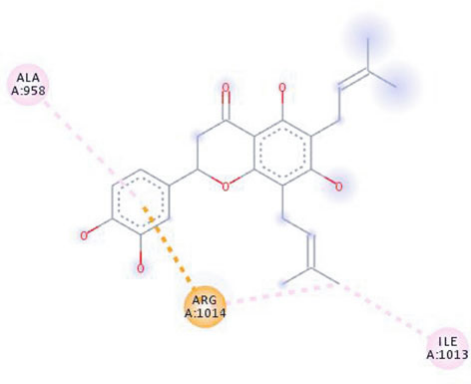
18.	Robustone	-9.0	0.249	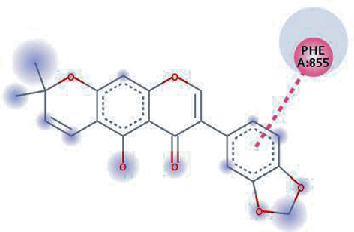
19.	Mundulinol	-9.0	0.249	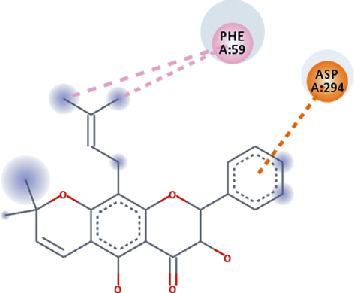
20.	Narlumicine	-8.9	0.294	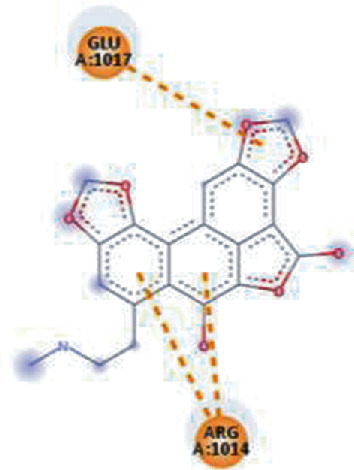
21.	Papracinine	-8.9	0.294	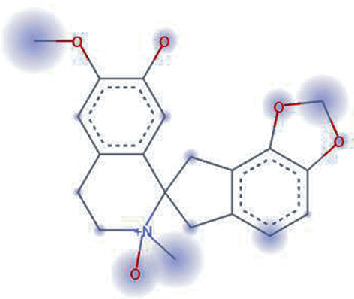
22.	Oxysanguinarine	-8.8	0.349	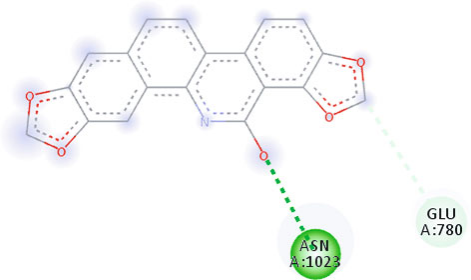
23.	Papraine	-8.8	0.349	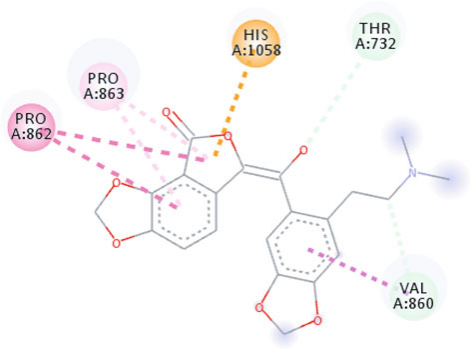
24.	Paprarine	-8.8	0.349	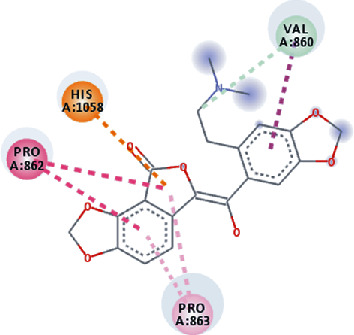
25.	AbyssinoneV	-8.8	0.349	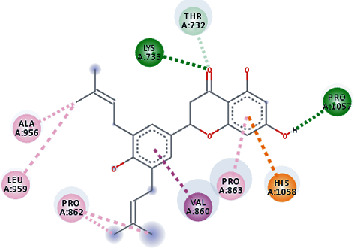
26.	TomentodiplaconeB	-8.8	0.349	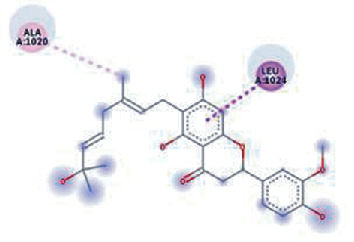
27.	Emodin	-8.7	0.413	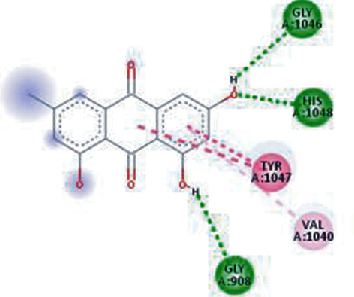
28.	SigmoidinB	-8.7	0.413	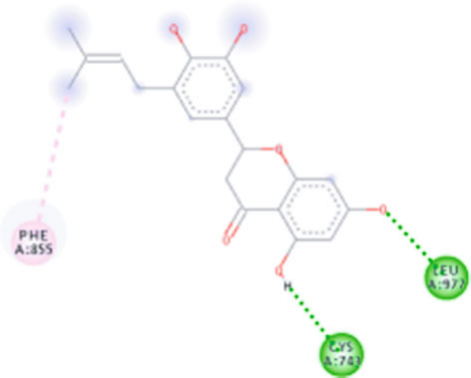
29.	SilybinB	-8.7	0.413	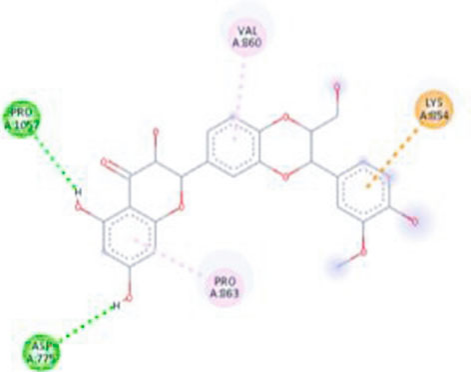
30.	IsosilybinB	-8.7	0.413	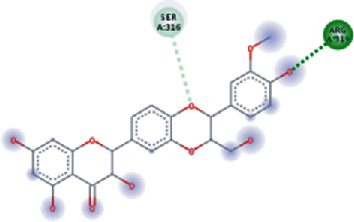
31.	SchizolaenoneB	-8.7	0.413	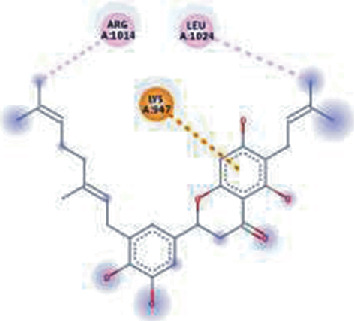
32.	EryvarinQ	-8.6	0.489	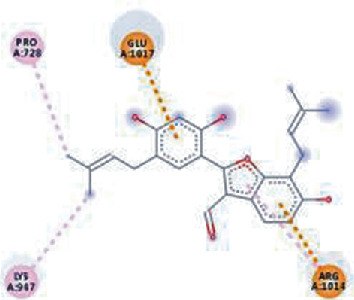
33.	IsoerysenegalenseinE	-8.6	0.489	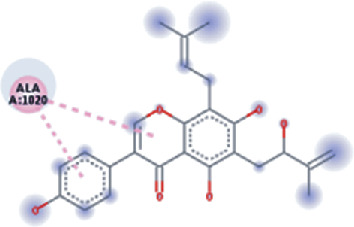
34.	Laburnetin	-8.6	0.489	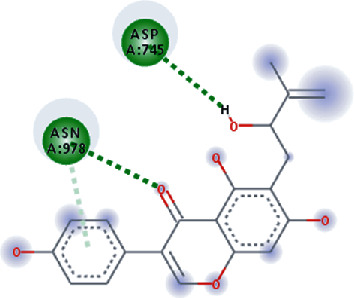
35.	Isomangostin	-8.6	0.489	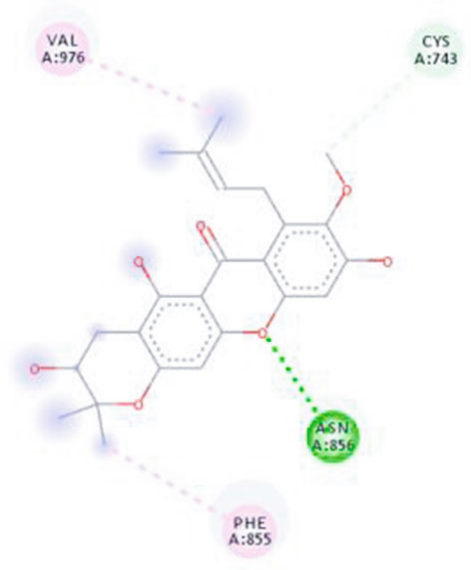
36.	Raddeanine	-8.5	0.579	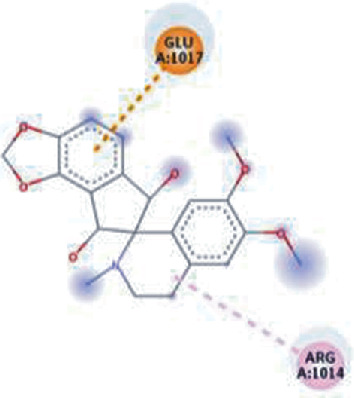

**Table 2:** Average RMSD values for all complexes at temperatures 300 K, 325 K, 340 K, and 350 K.

The complex of HR1 and phytochemicals	Average RMSD at various temperatures (Å)			
	300 K	325 K	340 K	350 K
SilybinC	0.55	0.77	1.25	1.36
Isopomiferin	0.59	1.04	1.29	1.61
Lycopene	1.20	1.70	1.89	1.96
SilydianinB	1.65	1.88	2.62	2.87
Silydianin	1.70	1.93	2.72	2.94

**Table 3:** Reactivity of phytochemicals with HR1 domain, depicted by band energy gaps.

The complex of HR1 domain and phytochemicals	*E*_LUMO_ (kcal/mol)	*E*_HOMO_ (kcal/mol)	Band energy gap (Δ*E*) (kcal/mol)
SilybinC	-0.280	-0.395	0.116
Isopomiferin	-0.218	-0.335	0.117
Lycopene	-0.229	-0.342	0.114
SilydianinB	-0.164	-0.288	0.124
Silydianin	-0.212	-0.339	0.127

## Data Availability

The data used to support the findings of this study are included within the supplementary information files.

## Supplementary Materials

Supplementary 1 Table S1: ADMET of screened 108 phytochemicals.

Supplementary 2 Table S2: list of 463 plants.

Supplementary 3 Table S3: binding affinities of 108 docked phytochemicals.
