# Supplementary material for: Virtual Screening of Phytochemicals by Targeting HR1 Domain of SARS-CoV-2 S Protein: Molecular Docking, Molecular Dynamics Simulations, and DFT Studies
Source: Biomed Res Int. 2021 May 20;2021:6661191. doi: 10.1155/2021/6661191 (PMC8139335; doi:10.1155/2021/6661191)
Supplement: Supplementary 1 — Table S1: ADMET of screened 108 phytochemicals. [file 6661191.f1.docx]

**Table S1:** ADMET of Screened 108 Phytochemicals

| **Phytochemicals** | **Plant Name** | **ESOL Class** | **GI Absorption** | **BBB Penetration** | **Lipinski violations** | **Toxicity** | **Carcinogenicity** |
| --- | --- | --- | --- | --- | --- | --- | --- |
| (+)-medioresinol | *Aloe Vera* | Soluble | High | No | 0 | Non-Toxic | Non-Carcinogenic |
| (+)-syringaresinol | *Aloe Vera* | Soluble | High | No | 0 | Non-Toxic | Non-Carcinogenic |
| 12S-hydroxyandrographolide | *Fumaria Indica* | Soluble | High | No | 0 | Non-Toxic | Non-Carcinogenic |
| 19-hydroxy-8 (17), 13-labdadien-15, 16-olide | *Fumaria Indica* | Soluble | High | No | 0 | Non-Toxic | Non-Carcinogenic |
| 3, 4-dihydroxhbenzoic acid | *Tanacetum Parthenium* | Soluble | High | No | 0 | Non-Toxic | Non-Carcinogenic |
| 3-methoxy-4-hydroxyienzoic acid | *Tanacetum Parthenium* | Soluble | High | No | 0 | Non-Toxic | Non-Carcinogenic |
| 3-oxo-14-deoxy-andrographolide | *Fumaria Indica* | Soluble | High | No | 0 | Non-Toxic | Non-Carcinogenic |
| 5, 7, 3', 4'-tetrahydroxyflavone | *Solanum nigrum* | Soluble | High | No | 0 | Non-Toxic | Non-Carcinogenic |
| 5, 7, 4'-trihydroxyflavone | *Solanum nigrum* | Soluble | High | No | 0 | Non-Toxic | Non-Carcinogenic |
| AbyssinoneV | *Erythrina Varigatae* | Soluble | High | No | 0 | Non-Toxic | Non-Carcinogenic |
| Adenosine | *Tanacetum Parthenium* | Soluble | High | No | 0 | Non-Toxic | Non-Carcinogenic |
| Aloe Emodin | *Aloe Vera* | Soluble | High | No | 0 | Non-Toxic | Non-Carcinogenic |
| Alpha Tetrapathe | *Tanacetum Parthenium* | Soluble | High | No | 0 | Non-Toxic | Non-Carcinogenic |
| AmyrisinA | *Silybum Marianum* | Soluble | High | No | 0 | Non-Toxic | Non-Carcinogenic |
| AmyrisinB | *Silybum Marianum* | Soluble | High | No | 0 | Non-Toxic | Non-Carcinogenic |
| AmyrisinC | *Silybum Marianum* | Soluble | High | No | 0 | Non-Toxic | Non-Carcinogenic |
| AndrographidoidsA | *Santolina insularis* | Soluble | High | No | 0 | Non-Toxic | Non-Carcinogenic |
| Anthraxin | *Silybum Marianum* | Soluble | High | No | 0 | Non-Toxic | Non-Carcinogenic |
| Apigenin | *Tanacetum Parthenium* | Soluble | High | No | 0 | Non-Toxic | Non-Carcinogenic |
| ApigeninB | *Solanum nigrum* | Soluble | High | No | 0 | Non-Toxic | Non-Carcinogenic |
| Ascorbic Acid | *Fumaria Indica* | Soluble | High | No | 0 | Non-Toxic | Non-Carcinogenic |
| Benzaldehyde | *Tanacetum Parthenium* | Soluble | High | No | 0 | Non-Toxic | Non-Carcinogenic |
| Caffeic Acid | *Solanum nigrum* | Soluble | High | No | 0 | Non-Toxic | Non-Carcinogenic |
| Cannflavin | *Silybum Marianum* | Soluble | High | No | 0 | Non-Toxic | Non-Carcinogenic |
| Cirsimaritin | *Santolina insularis* | Soluble | High | No | 0 | Non-Toxic | Non-Carcinogenic |
| Costinulide | *Tanacetum Parthenium* | Soluble | High | No | 0 | Non-Toxic | Non-Carcinogenic |
| Derrisin | *Silybum Marianum* | Soluble | High | No | 0 | Non-Toxic | Non-Carcinogenic |
| Dihydroxy-348-trimethoxyxanthone | *Silybum Marianum* | Soluble | High | No | 0 | Non-Toxic | Non-Carcinogenic |
| Diprenyleriodictyol | *Silybum Marianum* | Soluble | High | No | 0 | Non-Toxic | Non-Carcinogenic |
| DoitunggarcinoneC | *Silybum Marianum* | Soluble | High | No | 0 | Non-Toxic | Non-Carcinogenic |
| Emodin | *Tanacetum Parthenium* | Soluble | High | No | 0 | Non-Toxic | Non-Carcinogenic |
| Epoxy | *Fumaria Indica* | Soluble | High | No | 0 | Non-Toxic | Non-Carcinogenic |
| Erycristagallin | *Tamarix nilotica* | Soluble | High | No | 0 | Non-Toxic | Non-Carcinogenic |
| Erythrinins B | *Erythrina Varigatae* | Soluble | High | No | 0 | Non-Toxic | Non-Carcinogenic |
| EryvarinM | *Erythrina Varigatae* | Soluble | High | No | 0 | Non-Toxic | Non-Carcinogenic |
| EryvarinO | *Erythrina Varigatae* | Soluble | High | No | 0 | Non-Toxic | Non-Carcinogenic |
| EryvarinolsA | *Erythrina Varigatae* | Soluble | High | No | 0 | Non-Toxic | Non-Carcinogenic |
| EryvarinP | *Erythrina Varigatae* | Soluble | High | No | 0 | Non-Toxic | Non-Carcinogenic |
| EryvarinQ | *Erythrina Varigatae* | Soluble | High | No | 0 | Non-Toxic | Non-Carcinogenic |
| EryvarinR | *Erythrina Varigatae* | Soluble | High | No | 0 | Non-Toxic | Non-Carcinogenic |
| Estafin | *Fumaria Indica* | Soluble | High | No | 0 | Non-Toxic | Non-Carcinogenic |
| EuchrenoneB | *Erythrina Varigatae* | Soluble | High | No | 0 | Non-Toxic | Non-Carcinogenic |
| Feruloyltyramine | *Fumaria Indica* | Soluble | High | No | 0 | Non-Toxic | Non-Carcinogenic |
| Fumaric Acid | *Solanum nigrum* | Soluble | High | No | 0 | Non-Toxic | Non-Carcinogenic |
| Fumaritine N-oxide | *Fumaria Indica* | Soluble | High | No | 0 | Non-Toxic | Non-Carcinogenic |
| Hydroxy Erythratidine | *Erythrina Varigatae* | Soluble | High | No | 0 | Non-Toxic | Non-Carcinogenic |
| Hydroxyanhydro | *Fumaria Indica* | Soluble | High | No | 0 | Non-Toxic | Non-Carcinogenic |
| Hydroxygenistein | *Erythrina Varigatae* | Soluble | High | No | 0 | Non-Toxic | Non-Carcinogenic |
| Hydroxymunduserone | *Silybum Marianum* | Soluble | High | No | 0 | Non-Toxic | Non-Carcinogenic |
| Isoach | *Fumaria Indica* | Soluble | High | No | 0 | Non-Toxic | Non-Carcinogenic |
| IsoerysenegalenseinE | *Erythrina Varigatae* | Soluble | High | No | 0 | Non-Toxic | Non-Carcinogenic |
| Isomangostin | *Silybum Marianum* | Soluble | High | No | 0 | Non-Toxic | Non-Carcinogenic |
| Isopomiferin | *Silybum Marianum* | Soluble | High | No | 0 | Non-Toxic | Non-Carcinogenic |
| IsosilybinA | *Silybum Marianum* | Soluble | High | No | 0 | Non-Toxic | Non-Carcinogenic |
| IsosilybinB | *Silybum Marianum* | Soluble | High | No | 0 | Non-Toxic | Non-Carcinogenic |
| Laburnetin | *Erythrina Varigatae* | Soluble | High | No | 0 | Non-Toxic | Non-Carcinogenic |
| Lenticin | *Erythrina Varigatae* | Soluble | High | No | 0 | Non-Toxic | Non-Carcinogenic |
| Lupiwighteone | *Erythrina Varigatae* | Soluble | High | No | 0 | Non-Toxic | Non-Carcinogenic |
| Luteolin | *Melissa officinalis* | Soluble | High | No | 0 | Non-Toxic | Non-Carcinogenic |
| Lycopene | *Moringa peregrina* | Soluble | High | No | 0 | Non-Toxic | Non-Carcinogenic |
| Mearnsetin | *Silybum Marianum* | Soluble | High | No | 0 | Non-Toxic | Non-Carcinogenic |
| Menisdaurin | *Andrographis paniculata* | Soluble | High | No | 0 | Non-Toxic | Non-Carcinogenic |
| Mundulinol | *Silybum Marianum* | Soluble | High | No | 0 | Non-Toxic | Non-Carcinogenic |
| Myrcene | *Tamarix nilotica* | Soluble | High | No | 0 | Non-Toxic | Non-Carcinogenic |
| Narlumicine | *Tanacetum Parthenium* | Soluble | High | No | 0 | Non-Toxic | Non-Carcinogenic |
| Narlumidine | *Tanacetum Parthenium* | Soluble | High | No | 0 | Non-Toxic | Non-Carcinogenic |
| Noroxyhydrastinine | *Andrographis paniculata* | Soluble | High | No | 0 | Non-Toxic | Non-Carcinogenic |
| OphiopogonanoneG | *Silybum Marianum* | Soluble | High | No | 0 | Non-Toxic | Non-Carcinogenic |
| Osajin | *Erythrina Varigatae* | Soluble | High | No | 0 | Non-Toxic | Non-Carcinogenic |
| Oxyresveratrol | *Erythrina Varigatae* | Soluble | High | No | 0 | Non-Toxic | Non-Carcinogenic |
| Oxysanguinarine | *Tanacetum Parthenium* | Soluble | High | No | 0 | Non-Toxic | Non-Carcinogenic |
| Pantothenic Acid | *Fumaria Indica* | Soluble | High | No | 0 | Non-Toxic | Non-Carcinogenic |
| Papracinine | *Fumaria Indica* | Soluble | High | No | 0 | Non-Toxic | Non-Carcinogenic |
| Paprafumine | *Fumaria Indica* | Soluble | High | No | 0 | Non-Toxic | Non-Carcinogenic |
| Papraine | *Fumaria Indica* | Soluble | High | No | 0 | Non-Toxic | Non-Carcinogenic |
| Papraline | *Santolina insularis* | Soluble | High | No | 0 | Non-Toxic | Non-Carcinogenic |
| Paprarine | *Fumaria Indica* | Soluble | High | No | 0 | Non-Toxic | Non-Carcinogenic |
| Parthenolide | *Santolina insularis* | Soluble | High | No | 0 | Non-Toxic | Non-Carcinogenic |
| Pinocarvone | *Tamarix nilotica* | Soluble | High | No | 0 | Non-Toxic | Non-Carcinogenic |
| Potassium Sorbate | *Solanum nigrum* | Soluble | High | No | 0 | Non-Toxic | Non-Carcinogenic |
| Pyridoxine | *Santolina insularis* | Soluble | High | No | 0 | Non-Toxic | Non-Carcinogenic |
| Raddeanine | *Andrographis paniculata* | Soluble | High | No | 0 | Non-Toxic | Non-Carcinogenic |
| Rhamnetin | *Solanum nigrum* | Soluble | High | No | 0 | Non-Toxic | Non-Carcinogenic |
| Riboflavin | *Andrographis paniculata* | Soluble | High | No | 0 | Non-Toxic | Non-Carcinogenic |
| Robustone | *Erythrina Varigatae* | Soluble | High | No | 0 | Non-Toxic | Non-Carcinogenic |
| SchizolaenoneB | *Silybum Marianum* | Soluble | High | No | 0 | Non-Toxic | Non-Carcinogenic |
| Sesquiterpene Glycoside | *Tanacetum Parthenium* | Soluble | High | No | 0 | Non-Toxic | Non-Carcinogenic |
| SigmoidinA | *Erythrina Varigatae* | Soluble | High | No | 0 | Non-Toxic | Non-Carcinogenic |
| SigmoidinB | *Erythrina Varigatae* | Soluble | High | No | 0 | Non-Toxic | Non-Carcinogenic |
| SigmoidinC | *Erythrina Varigatae* | Soluble | High | No | 0 | Non-Toxic | Non-Carcinogenic |
| SilybinA | *Silybum Marianum* | Soluble | High | No | 0 | Non-Toxic | Non-Carcinogenic |
| SilybinB | *Silybum Marianum* | Soluble | High | No | 0 | Non-Toxic | Non-Carcinogenic |
| SilybinC | *Silybum Marianum* | Soluble | High | No | 0 | Non-Toxic | Non-Carcinogenic |
| SilybinD | *Silybum Marianum* | Soluble | High | No | 0 | Non-Toxic | Non-Carcinogenic |
| Silydianin | *Silybum Marianum* | Soluble | High | No | 0 | Non-Toxic | Non-Carcinogenic |
| silydianinB | *Silybum Marianum* | Soluble | High | No | 0 | Non-Toxic | Non-Carcinogenic |
| Stachydrine | *Erythrina Varigatae* | Soluble | High | No | 0 | Non-Toxic | Non-Carcinogenic |
| Tamarixetin | *Tamarix nilotica* | Soluble | High | No | 0 | Non-Toxic | Non-Carcinogenic |
| Tanaparthe | *Solanum nigrum* | Soluble | High | No | 0 | Non-Toxic | Non-Carcinogenic |
| Tanaparthe | *Solanum nigrum* | Soluble | High | No | 0 | Non-Toxic | Non-Carcinogenic |
| Tanetin | *Santolina insularis* | Soluble | High | No | 0 | Non-Toxic | Non-Carcinogenic |
| TanetinB | *Silybum Marianum* | Soluble | High | No | 0 | Non-Toxic | Non-Carcinogenic |
| Taxifolin | *Silybum Marianum* | Soluble | High | No | 0 | Non-Toxic | Non-Carcinogenic |
| Thiamine | *Andrographis paniculata* | Soluble | High | No | 0 | Non-Toxic | Non-Carcinogenic |
| TomentodiplaconeB | *Silybum Marianum* | Soluble | High | No | 0 | Non-Toxic | Non-Carcinogenic |
| Uric Acid | *Andrographis paniculata* | Soluble | High | No | 0 | Non-Toxic | Non-Carcinogenic |
| Wighteone | *Erythrina Varigatae* | Soluble | High | No | 0 | Non-Toxic | Non-Carcinogenic |
| Xylan | *Tanacetum Parthenium* | Soluble | High | No | 0 | Non-Toxic | Non-Carcinogenic |
