# Supplementary material for: Virtual Screening of Phytochemicals by Targeting HR1 Domain of SARS-CoV-2 S Protein: Molecular Docking, Molecular Dynamics Simulations, and DFT Studies
Source: Biomed Res Int. 2021 May 20;2021:6661191. doi: 10.1155/2021/6661191 (PMC8139335; doi:10.1155/2021/6661191)
Supplement: Supplementary 2 — Table S2: list of 463 plants. [file 6661191.f2.docx]

**Table S2:** List of 463 Plants

| **Plant Name** | **Plant Name** | **Plant Name** |
| --- | --- | --- |
| Abrus precatorius | Datura stramonium | Mimosa pudica |
| Acalypha indica | Decalepis hamiltonii | Mitragyna parvifolia |
| Acanthus ilicifolius | Delphinium brunonianum | Momordica charantia |
| Achillea millefolium | Delphinium caeruleum | Moringa oleifera |
| Aconitum chasmanthum | Delphinium cashmirianum | Moringa peregrina |
| Aconitum deinorrhizum | Delphinium denudatum | Morus alba |
| Aconitum falconeri | Delphinium elatum | Mucuna pruriens |
| Aconitum ferox | Delphinium nordhagenii | Murraya koenigii |
| Aconitum heterophyllum | Delphinium semibarbatum | Murraya paniculata |
| Aconitum laeve | Delphinium vestitum | Musa sapientum |
| Aconitum napellus | Dendrobium nobile | Naravelia zeylanica |
| Aconitum spicatum | Desmodium pulchellum | Narcissus tazetta |
| Aconitum violaceum | Desmodium styracifolium | Nauclea orientalis |
| Acronychia pedunculata | Desmodium tiliaefolium | Nelumbo nucifera |
| Aegle marmelos | Desmodium triflorum | Nepeta cataria |
| Aerva lanata | Desmos chinensis | Nicotiana tabacum |
| Aglaia odorata | Dianthus caryophyllus | Nigella damascena |
| Ailanthus altissima | Dicentra chrysantha | Nigella sativa |
| Ailanthus excelsa | Dichroa febrifuga | Nymphaea alba |
| Alhagi pseudalhagi | Dictamnus albus | Olea paniculata |
| Alkanna tinctoria | Dioscorea hispida | Ophiorrhiza mungos |
| Alliaria petiolata | Drymaria cordata | Oryza sativa |
| Allium chinense | Drymaria diandra | Pachygone ovata |
| Allium tuberosum | Echinops echinatus | Panax ginseng |
| Aloe Vera | Elaeagnus angustifolia | Papaver argemone |
| Alseodaphne semicarpifolia | Elaeocarpus sphaericus | Papaver dubium |
| Alstonia constricta | emia dissecta | Papaver nudicaule |
| Alstonia macrophylla | Entada phaseoloides | Papaver orientale |
| Alstonia scholaris | Entada pursaetha | Papaver pavoninum |
| Alstonia spectabilis | Ephedra intermedia | Papaver rhoeas |
| Alstonia venenata | Ephedra sinica | Papaver somniferum |
| Amaranthus tricolor | Ervatamia heyneana | Parsonsia laevigata |
| Amphicome emodi | Ervatamia orientalis | Passiflora edulis |
| Anacyclus pyrethrum | Erythrina arborescens | Pedicularis longiflora |
| Anamirta cocculus | Erythrina fusca | Peganum harmala |
| Ananas comosus | Erythrina stricta | Perilla frutescens |
| Ancistrocladus heyneanus | Erythrina suberosa | Peristrophe roxburghiana |
| Ancistrocladus tectorius | Erythrina variegata | Phalaris arundinacea |
| Andrachne aspera | Erythrina Varigatae | Phoenix dactylifera |
| Andrographis paniculata | Erythrophleum africanum | Phyllagathis rotundifolia |
| Annona cherimola | Erythroxylum coca | Phyllanthus amarus |
| Annona muricata | Erythroxylum lucidum | Phyllanthus fraternus |
| Annona squamosa | Erythroxylum monogynum | Physalis alkekengi |
| Antirrhinum majus | Euphorbia antiquorum | Physalis peruviana |
| Arachis hypogaea | Euphorbia atoto | Physostigma venenosum |
| Argemone mexicana | Euphorbia cornigera | Picea abies |
| Argyreia cuneata | Euphorbia lathyris | Picrasma javanica |
| Argyreia nervosa | Euphorbia milii | Picrasma quassioides |
| Aristolochia indica | Euphorbia nivulia | Pimpinella anisum |
| Aristolochia longa | Euphorbia resinifera | Piper argyrophyllum |
| Aristolochia rotunda | Euphorbia tirucalli | Piper guineense |
| Aristolochia serpentaria | Eurycoma longifolia | Piper longum |
| Aristolochia tagala | Evodia lunu-ankenda | Piper nigrum |
| Arnica montana | Evodia meliaefolia | Piper peepuloides |
| Artabotrys suaveolens | Evodia rutaecarpa | Piper retrofractum |
| Artemisia dracunculus | Fabiana imbricata | Piper sarmentosum |
| Artemisia rutifolia | Fagopyrum esculentum | Piper sylvaticum |
| Atalantia monophylla | Fibraurea chloroleuca | Piper trichostachyon |
| Atropa belladonna | Fibraurea tinctoria | Pisum sativum |
| Avena sativa | Ficus microcarpa | Plantago Ovata |
| Azima tetracantha | Foeniculum vulgare | Pleiospermium alatum |
| Bambusa vulgaris | Fritillaria imperialis | Pleurostylia opposita |
| Basella rubra | Fumaria Indica | Polyalthia longifolia |
| Berberis lamberti | Fumaria officinalis | Portulaca grandiflora |
| Berberis lycium | Fumaria vaillantii | Portulaca oleracea |
| Berberis orthobotrys | Galega officinalis | Pratia nummularia |
| Berberis vulgaris | Gaultheria nummularioides | Prosopis juliflora |
| Beta vulgaris | Gelsemium elegans | Prunus persica |
| Boenninghausenia albiflora | Gentiana olivieri | Psychotria ipecacuanha |
| Bongardia chrysogonum | Gloriosa superba | Punica granatum |
| Brassica campestris | Glycine max | Pyrus communis |
| Brassica juncea | Glycosmis arborea | Raphanus sativus |
| Brassica napus | Glycosmis cochinchinensis | Reseda odorata |
| Brassica oleracea | Glycosmis mauritiana | Rhaphidophora decursiva |
| Brassica rapa | Glycosmis pentaphylla | Rhazya stricta |
| Brucea mollis | Glycyrrhiza uralensis | Rhizophora mucronata |
| Bruguiera cylindrica | Gomphrena globosa | Ribes nigrum |
| Butea monosperma | Gossypium hirsutum | Ribes rubrum |
| Buxus sempervirens | Gyrocarpus americanus | Ricinus communis |
| Buxus wallichiana | Haemanthus multiflorus | Rorippa montana |
| Caesalpinia digyna | Hamelia patens | Rosmarinus officinalis |
| Camellia sinensis | Haplophyllum tuberculatum | Rubia cordifolia |
| Cananga odorata | Hedera helix | Ruta chalepensis |
| Canavalia gladiata | Hedyotis capitellata | Ruta graveolens |
| Cannabis sativa | Helianthus annuus | Salvadora persica |
| Capparis decidua | Heliotropium curassavicum | Sambucus nigra |
| Capparis himalayensis | Heliotropium europaeum | Santalum album |
| Capsicum annuum | Heliotropium indicum | Santolina insularis |
| Capsicum frutescens | Heliotropium ovalifolium | Sapium sebiferum |
| Carallia brachiata | Heliotropium scabrum | Sarcococca saligna |
| Cardiospermum halicacabum | Heliotropium subulatum | Schizophyllum commune |
| Carica papaya | Heliotropium supinum | Securinega suffruticosa |
| Carthamus tinctorius | Helleborus viridis | Securinega virosa |
| Cassia absus | Hemerocallis fulva | Sida cordifolia |
| Cassia siamea | Hernandia ovigera | Silybum Marianum |
| Cassytha filiformis | Hibiscus cannabinus | Sinapis alba |
| Castanea sativa | Hibiscus sabdariffa | Skimmia laureola |
| Castanospermum australe | Hibiscus syriacus | Solanum giganteum |
| Catha edulis | Holarrhena mitis | Solanum nigrum |
| Catharanthus roseus | Holarrhena pubescens | Solanum pubescens |
| Celosia argentea | Hordeum jubatum | Solanum tuberosum |
| Celosia cristata | Hordeum vulgare | Sophora griffithii |
| Chenopodium album | Houttuynia cordata | Sophora tomentosa |
| Chonemorpha fragrans | Hunteria umbellata | Sorghum bicolor |
| Cicer arietinum | Hunteria zeylanica | Spilanthes acmella |
| Cinchona calisaya | Hyoscyamus niger | Spilanthes oleracea |
| Cinchona ledgeriana | Hypecoum leptocarpum | Stachys sylvatica |
| Cinchona succirubra | Hypecoum procumbens | Stellaria aquatica |
| Cirsium arvense | Indigofera tinctoria | Stemona tuberosa |
| Cissampelos pareira | Iphigenia indica | Stephania glabra |
| Citrus grandis | Ipomoea alba | Stephania japonica |
| Citrus paradisi | Ipomoea hederifolia | Stephania rotunda |
| Citrus sinensis | Ipomoea quamoclit | Stictocardia campanulata |
| Clausena excavata | Ipomoea violacea | Strychnos cinnamomifolia |
| Clausena heptaphylla | Isatis tinctoria | Strychnos gaultheriana |
| Clausena lansium | Isopyrum thalictroides | Strychnos ignatii |
| Claviceps purpurea | Jateorhiza palmata | Strychnos nux-vomica |
| Cocculus hirsutus | Kopsia flavida | Strychnos potatorum |
| Cocculus laurifolius | Kopsia fruticosa | Strychnos wallichiana |
| Cocculus pendulus | Lagerstroemia indica | Tabernaemontana dichotoma |
| Cocos nucifera | Lathyrus odoratus | Tabernaemontana divaricata |
| Codonopsis clematidea | Lathyrus sativus | Tamarix nilotica |
| Coffea arabica | Laurus nobilis | Tanacetum Parthenium |
| Colchicum autumnale | Lens culinaris | Tecoma stans |
| Colchicum luteum | Leonurus sibiricus | Thalictrum alpinum |
| Commelina communis | Lepidium sativum | Thalictrum cultratum |
| Conium maculatum | Leucaena leucocephala | Thalictrum foetidum |
| Consolida ambigua | Leuconotis eugenifolia | Thalictrum minus |
| Consolida regalis | Lilium candidum | Thalictrum pedunculatum |
| Conyza bonariensis | Lilium martagon | Theobroma cacao |
| Corydalis govaniana | Limonia acidissima | Toddalia asiatica |
| Corydalis meifolia | Linum usitatissimum | Tribulus terrestris |
| Corydalis vaginans | Liparis nervosa | Trifolium pratense |
| Coscinium fenestratum | Liriodendron tulipifera | Trifolium repens |
| Crinum asiaticum | Lithospermum officinale | Triglochin maritima |
| Crinum latifolium | Litsea cubeba | Trigonella foenum-graecum |
| Crinum powellii | Litsea glutinosa | Triticum aestivum |
| Crotalaria albida | Lobelia nicotianaefolia | Tylophora hirsuta |
| Crotalaria burhia | Lupinus albus | Tylophora indica |
| Crotalaria laburnifolia | Lupinus angustifolius | Uncaria gambier |
| Crotalaria mucronata | Lupinus digitatus | Valeriana officinalis |
| Crotalaria retusa | Lupinus luteus | Vepris bilocularis |
| Crotalaria spectabilis | Lycium barbarum | Vicia faba |
| Crotalaria striata | Lycopersicon esculentum | Vicia sativa |
| Croton tiglium | Lycopodium clavatum | Vinca major |
| Cucubalus baccifer | Lycoris radiata | Viscum album |
| Cucurbita pepo | Mangifera indica | Withania somnifera |
| Cuscuta reflexa | Marrubium vulgare | Xylocarpus granatum |
| Cycas circinalis | Maytenus emarginata | Zanthoxylum nitidum |
| Cycas revoluta | Maytenus senegalensis | Zanthoxylum ovalifolium |
| Cynanchum vincetoxicum | Medicago sativa | Zanthoxylum rhetsa |
| Cyperus esculentus | Melilotus officinalis | Zea mays |
| Cytisus scoparius | Melissa officinalis | Ziziphus oenoplia |
| Datura innoxia | Melochia corchorifolia | Zizyphus spinachristi |
| Datura metel |  |  |
