# Supplementary material for: Virtual Screening of Phytochemicals by Targeting HR1 Domain of SARS-CoV-2 S Protein: Molecular Docking, Molecular Dynamics Simulations, and DFT Studies
Source: Biomed Res Int. 2021 May 20;2021:6661191. doi: 10.1155/2021/6661191 (PMC8139335; doi:10.1155/2021/6661191)
Supplement: Supplementary 3 — Table S3: binding affinities of 108 docked phytochemicals. [file 6661191.f3.docx]

**Table S3:** Binding affinities of 108 docked phytochemicals

| **S.No.** | **Phytochemicals** | **Binding affinity (kcal/mol)** |
| --- | --- | --- |
|  | SilybinC | -10.0 |
|  | Isopomiferin | -9.9 |
|  | Lycopene | -9.8 |
|  | SilydianinB | -9.8 |
|  | Silydianin | -9.7 |
|  | Anthraxin | -9.5 |
|  | Derrisin | -9.5 |
|  | Osajin | -9.5 |
|  | SigmoidinA | -9.4 |
|  | SigmoidinC | -9.4 |
|  | EuchrenoneB | -9.3 |
|  | SilybinD | -9.3 |
|  | Cannflavin | -9.2 |
|  | IsosilybinA | -9.2 |
|  | Diprenyleriodictyol | -9.1 |
|  | Fumaritine N-oxide | -9.1 |
|  | SilybinA | -9.1 |
|  | Mundulinol | -9.0 |
|  | Robustone | -9.0 |
|  | Narlumicine | -8.9 |
|  | Papracinine | -8.9 |
|  | AbyssinoneV | -8.8 |
|  | Oxysanguinarine | -8.8 |
|  | Papraine | -8.8 |
|  | Paprarine | -8.8 |
|  | TomentodiplaconeB | -8.8 |
|  | Emodin | -8.7 |
|  | IsosilybinB | -8.7 |
|  | SchizolaenoneB | -8.7 |
|  | SigmoidinB | -8.7 |
|  | SilybinB | -8.7 |
|  | EryvarinQ | -8.6 |
|  | IsoerysenegalenseinE | -8.6 |
|  | Isomangostin | -8.6 |
|  | Laburnetin | -8.6 |
|  | Raddeanine | -8.5 |
|  | 3, 4-dihydroxhbenzoic acid | -7.9 |
|  | Benzaldehyde | -7.8 |
|  | Epoxy | -7.8 |
|  | Menisdaurin | -7.8 |
|  | Rhamnetin | -7.8 |
|  | 3-methoxy-4-hydroxyienzoic acid | -7.6 |
|  | Noroxyhydrastinine | -7.6 |
|  | Potassium Sorbate | -7.6 |
|  | Tanetin | -7.6 |
|  | EryvarinO | -7.4 |
|  | Fumaric Acid | -7.4 |
|  | Tanaparthe | -7.4 |
|  | Alpha Tetrapathe | -7.3 |
|  | Ascorbic Acid | -7.3 |
|  | Hydroxygenistein | -7.3 |
|  | Isoach | -7.3 |
|  | AndrographidoidsA | -7.2 |
|  | AmyrisinC | -7.1 |
|  | EryvarinM | -7.1 |
|  | 5, 7, 4'-trihydroxyflavone | -7.0 |
|  | Myrcene | -7.0 |
|  | Taxifolin | -6.8 |
|  | 3-oxo-14-deoxy-andrographolide | -6.7 |
|  | Parthenolide | -6.7 |
|  | Dihydroxy-348-trimethoxyxanthone | -6.6 |
|  | Sesquiterpene Glycoside | -6.6 |
|  | Thiamine | -6.6 |
|  | Caffeic Acid | -6.5 |
|  | Feruloyltyramine | -6.5 |
|  | Pyridoxine | -6.5 |
|  | Riboflavin | -6.4 |
|  | Lupiwighteone | -6.3 |
|  | Luteolin | -6.3 |
|  | Adenosine | -6.2 |
|  | Xylan | -6.2 |
|  | Cirsimaritin | -6.1 |
|  | Tamarixetin | -6.1 |
|  | Paprafumine | -6.0 |
|  | Stachydrine | -6.0 |
|  | 5, 7, 3', 4'-tetrahydroxyflavone | -5.9 |
|  | Estafin | -5.9 |
|  | DoitunggarcinoneC | -5.8 |
|  | Erythrinins B | -5.8 |
|  | EryvarinP | -5.8 |
|  | Hydroxy Erythratidine | -5.8 |
|  | Hydroxyanhydro | -5.8 |
|  | OphiopogonanoneG | -5.8 |
|  | ApigeninB | -5.7 |
|  | Pinocarvone | -5.7 |
|  | AmyrisinA | -5.6 |
|  | Wighteone | -5.6 |
|  | (+)-syringaresinol | -5.5 |
|  | Mearnsetin | -5.4 |
|  | Uric Acid | -5.3 |
|  | TanetinB | -5.2 |
|  | Apigenin | -4.9 |
|  | EryvarinR | -4.9 |
|  | Narlumidine | -4.9 |
|  | 19-hydroxy-8 (17), 13-labdadien-15, 16-olide | -4.7 |
|  | Costinulide | -4.7 |
|  | Hydroxymunduserone | -4.7 |
|  | Pantothenic Acid | -4.7 |
|  | Oxyresveratrol | -4.6 |
|  | 12S-hydroxyandrographolide | -4.5 |
|  | Aloe Emodin | -4.5 |
|  | Erycristagallin | -4.5 |
|  | Lenticin | -4.5 |
|  | Tanaparthe | -4.5 |
|  | AmyrisinB | -4.3 |
|  | EryvarinolsA | -4.3 |
|  | (+)-medioresinol | -4.2 |
|  | Papraline | -4.2 |
